# Gentle stroking stimuli induce affiliative responsiveness to humans in male rats

**DOI:** 10.1038/s41598-020-66078-7

**Published:** 2020-06-04

**Authors:** Shota Okabe, Yuki Takayanagi, Masahide Yoshida, Tatsushi Onaka

**Affiliations:** Division of Brain and Neurophysiology, Department of Physiology, Jichi Medical University, 3311-1 Yakushiji, Shimotsuke-shi Tochigi-ken, 329-0498 Japan

**Keywords:** Emotion, Social behaviour

## Abstract

Gentle tactile stimuli have been shown to play an important role in the establishment and maintenance of affiliative social interactions. Oxytocin has also been shown to have similar actions. We investigated the effects of gentle stroking on affiliative relationships between humans and rats and the effects of gentle stroking on activation of oxytocin neurons. Male rats received 5-min stroking stimuli from an experimenter every other day for 4 weeks between 3 and 6 weeks of age (S3–6 group), for 4 weeks between 7 and 10 weeks of age (S7–10 group), or for 8 weeks between 3 and 10 weeks of age (S3–10 group). Control rats did not receive stroking stimuli. Rats in the S7–10 and S3–10 groups emitted 50-kHz calls, an index of positive emotion, more frequently during stroking stimuli. Rats in the S3–6, S7–10, and S3–10 groups showed affiliative behaviors toward the experimenter. Oxytocin neurons in the hypothalamic paraventricular nucleus of rats in the S3–6, S7–10, and S3–10 groups were activated following stroking stimuli. These findings revealed that post-weaning repeated stroking stimuli induce an affiliative relationship between rats and humans and activation of oxytocin neurons.

## Introduction

Social mammals develop affiliative relationships with intraspecific individuals. Affiliative relationships between mothers and infants^1,2^ and between pairs of males and females in monogamous species^3,4^ have been intensively investigated. Social animals sometimes establish affiliative relationships not only with intraspecific animals but also with interspecific animals. Sheep^5^, lambs^6^, and dogs^7^ have been shown to be able to establish affiliative relationships with humans. Other animals such as rats^8^, foxes^9^, dolphins^10^, cattle^11^, and horses^12^ have also been reported to show positive reactions toward humans. Affiliative interactions with animals have been suggested to reduce anxiety and to have positive effects on the health conditions of humans^13,14^. In addition, human-animal relationships improve the welfare of animals^15,16^. However, little is known about the neural mechanisms underlying the development and maintenance of human-animal affiliative relationships.

Rats emit several types of ultrasonic vocalizations (USVs). In aversive situations, such as exposure to predators, rats emit USVs with a frequency range of 18–32 kHz, referred to as “22-kHz calls”^17–21^. On the other hand, in appetitive situations, such as social play, rats elicit vocalizations with higher frequency (more than 35 kHz), referred to as “50-kHz calls”^20–22^. Thus, 22-kHz and 50-kHz calls are generally considered to be indicators of negative and positive emotional states, respectively^20^. Fifty-kHz calls have been further divided into frequency-modulated calls (FM calls) and flat calls (Non-FM calls). FM calls are further divided into several subtypes, including complex, upward, step up, and trill calls^20,23^. FM 50-kHz calls have been shown to be emitted during intensively affective situations such as social play and mating behavior^24^. In contrast, non-FM 50-kHz calls have been shown to be emitted during non-social situations such as feeding behavior^25^. These behavioral characteristics are useful for clarifying affiliative relationships between humans and rats in experimental situations. Thus, we used rats as good model animals for investigating heterogeneous affiliative relationships.

Tactile stimulation has been suggested to play a fundamental role in the establishment and maintenance of intimate relationships between individuals. Positive physical contacts such as hugging and massage by partners have been shown to reduce cortisol release and heart rate increase in response to stressful stimuli in humans^26^. In primates, social grooming has been suggested to facilitate affiliative bonds between individuals^27,28^. Not only conspecific grooming but also interspecific gentle stroking has been suggested to induce anxiolytic actions in behavioral and neuroendocrine systems in lambs^6^. We have also shown that massage-like gentle stroking stimuli induce 50-kHz calls in rats^29^. However, it was not known whether stroking stimuli induce an affiliative relationship between rats and humans. The amount and time period of stroking stimuli required to induce an affiliative response were also unknown.

Oxytocin is mainly synthesized in the hypothalamic paraventricular nucleus (PVN), supraoptic nucleus (SON), and bed nucleus of the stria terminalis (BNST). Oxytocin has been shown to facilitate affiliative social behaviors^30–33^ and to induce anti-stress or anxiolytic actions^34,35^. Several studies revealed that massage or non-noxious tactile stimulation increases oxytocin release in rats^29,36^, dogs^37^, and humans^38^. Massage-like hand movement of a baby induces an increase in plasma oxytocin concentrations in the mother^39^. We have also demonstrated that stroking stimuli activate oxytocin neurons, particularly in the caudal PVN^29^. From these findings, we considered that stroking stimuli activate oxytocin neurons and contribute to an affiliative relationship between humans and rats.

The present study aimed to clarify the effects of stroking stimuli on affiliative relationships between humans and rats and to determine the appropriate timing of stroking stimuli to create an affiliative relationship between humans and rats. For that purpose, male rats at the age of 3 weeks were assigned to four experimental groups with different periods of stroking, and then they received several behavioral tests for examination of anxiety-related behavior and affiliative behaviors toward an experimenter. Plasma testosterone concentrations, which affect social behaviors^40^, start to increase steeply at postnatal 7 weeks of age and reach adult concentrations at 9–10 weeks of age^41^. Thus, in the present study, stroking stimuli were applied during the postnatal periods of 3–6, 3–10, and 7–10 weeks. Effects of stroking stimuli on activation of oxytocin neurons were also investigated by examining the expression of c-Fos protein, an index of neuronal activation, in the hypothalamus and BNST.

## Materials and Methods

### Subjects

Animal experiments were carried out after receiving approval from the Animal Experiment Committee of Jichi Medical University and were in accordance with the Institutional Regulations for Animal Experiments and Fundamental Guidelines for Proper Conduct of Animal Experiments and Related Activities in Academic Research Institutions under the jurisdiction of the Ministry of Education, Culture, Sports, Science and Technology.

Forty-four male rats of the Lewis strain were used in the present study. These animals were produced by mating of pairs of sires and dams obtained from an animal supplier (LEW/ CrlCrlj, Charles River Laboratories Japan, Inc., Kanagawa, Japan) and were weaned at the age of three weeks. After weaning, male rats were housed in pairs and used in the present study. The animals were maintained under a 12: 12 h light/dark cycle (lights on at 7:30 am) at 22 ± 2 °C and 55 ± 15% relative humidity. Food and water were available *ad libitum*.

### Groups and general experimental design

**Figure 1:**
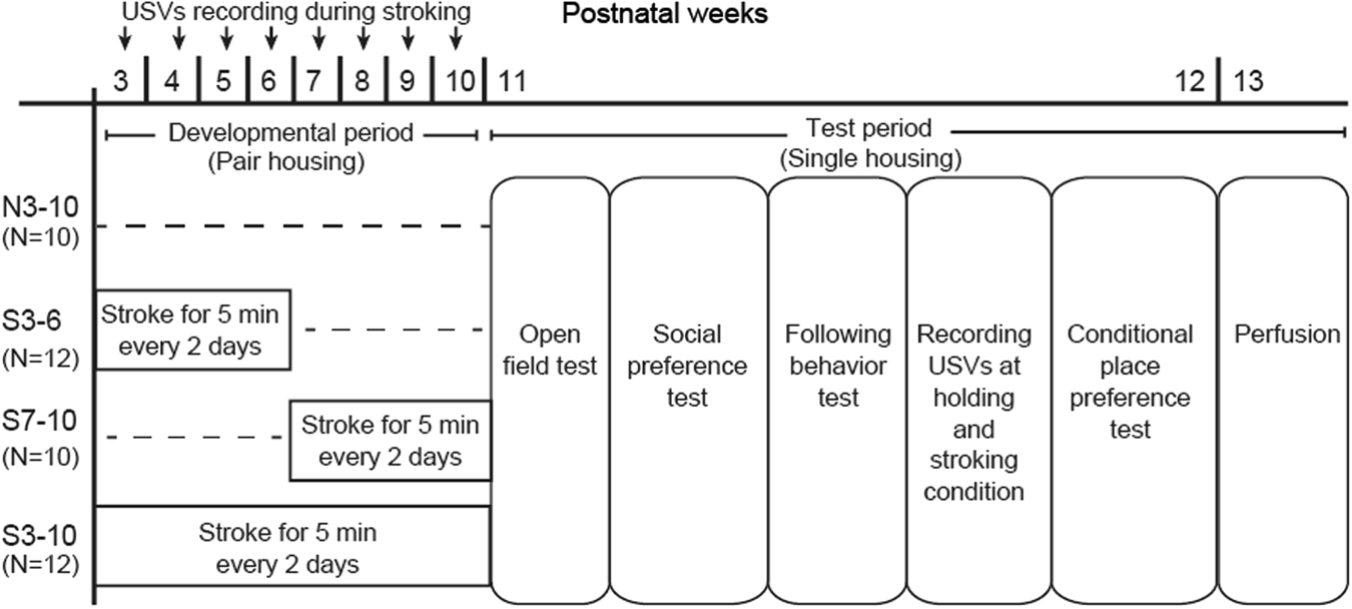
Time schedules of experiments. Ultrasonic vocalizations were recorded 5 min once per week between 3 and 10 weeks of age (developmental period) in the S3–6, S7–10, S3–10 groups. Behavioral tests were subsequently conducted in succession at the ages of 11 weeks and 12 weeks (test period).

Stroking stimuli were applied by an experimenter (S.O.) with the hand of the experimenter over the back of each animal at a speed of 5–10 cm/sec for 5 min every other day, as described previously^29^. Rats were assigned to four groups: S3–6 group, S7–10 group, S3–10 group, and N3–10 group. Rats in the S3–6 group received 5-min stroking stimuli from the experimenter for 4 weeks between 3 and 6 weeks of age (n = 12). Animals in the S7–10 group received stroking stimuli for 4 weeks between 7 and 10 weeks of age (n = 10). Animals in the S3–10 group received stroking stimuli for 8 weeks between 3 and 10 weeks of age (n = 12). Control rats in the N3–10 group did not receive stroking stimuli (n = 10). At 10 weeks of age, all of the rats were housed individually and then behavioral tests were conducted. Time schedules of treatments and behavioral tests are shown in Fig. 1.

### Ultrasonic vocalizations

#### General protocols for recording

Ultrasonic vocalizations (USVs) were recorded by use of a microphone (Type 4158 N, Aco, Tokyo, Japan) designed for measurements of sound pressure levels of 20–100,000 Hz sounds. The microphone was placed at a distance of approximately 20 cm from a rat and was connected to a sensor amplifier (SR-2200, Ono Sokki, Kanagawa, Japan). Acoustic data were recorded at a sampling rate of 200 kHz and with 16 bits by an Avisoft RECORDER (Version 4.2, Avisoft Bioacoustics, Berlin, Germany) and analyzed by a software program of Avisoft SASLab Pro (Version 5.2, Avisoft Bioacoustics). Spectrograms were generated with a fast Fourier transform length of 256 points and an overlap of 75%.

#### Recording of USVs

In the S3–6, S7–10, and S3–10 groups, USVs were recorded during a 5-min stroking session once each week during the developmental period (Fig. 1). Each rat on the experimenter’s lap was given massage-like stroking stimuli from the hand of the experimenter (S.O.). The numbers of 50-kHz calls (frequencies between 35 and 100 kHz) and 22-kHz calls (frequencies between 18 or more and less than 32 kHz) were manually counted.

During the test period, USVs in the basal condition when rats were kept in their individual home cages were recorded for 5 min more than 25 min after setting microphones above mesh lids of their home cages. USVs in a holding condition were recorded during a 5-min period when each rat was placed on the experimenter (S.O.)’s lap. USVs in a stroking condition were then recorded during the next 5-min stroking period. The numbers of 50-kHz calls and 22-kHz calls were manually counted. Fifty-kHz calls were divided into two types: FM and Non-FM calls. FM calls were further divided into 10 types: complex, upward, downward, split, step up, step down, multi-step, trill, inverted-U, and composite^23^. Non-FM calls were divided into two types: flat and short^23^. Fifty-kHz calls were manually classified into 12 types according to the shape of a spectrogram plot of each syllable. The numbers of these types of USVs were counted in basal, holding, and stroking conditions.

### Behavioral tests

#### Open field test

Anxiety-related behavior was analyzed by an open field test. Each animal was placed in an open field (W60 × D60 × H40 cm) and kept there for 10 min. Total movement distance was recorded as an index of locomotion, and total time spent staying in the center area, which was 36% of the total open field arena, and total time spent immobile were automatically measured by using a behavior analyzing program, Time OFCR1 (O’Hara & CO., LTD, Tokyo, Japan).

#### Social preference test

Preference to the experimenter (S.O.)’s hand compared with a novel object was determined by a social preference test. The experimenter (S.O.)’s hand was placed at one corner of a test box (W60 × D60 × H40 cm), and a novel plastic bottle (W6× D6 × H14 cm) was placed at the diagonally opposite corner of the test box. Each animal was introduced into the test box at a corner other than the corners where the hand and the bottle were placed. The time spent staying in a square (20 × 20 cm) where the hand or the bottle was placed was measured automatically during a 10-min test period by using an analyzing program (Time OFCR1). Preference score was calculated as [(time spent staying in the hand area) – (time spent staying in the bottle area)], and preference scores were compared among the four groups.

#### Following behavior test

Proactive approach behavior toward the experimenter (S.O.)’s hand (“following” behavior) was examined in a test box (W60 × D60 × H40 cm). The experimenter (S.O.)’s hand was placed at one corner of the test box. Each animal was placed at the corner diagonally opposite to the hand. The hand was moved from one corner to the next corner along the wall over a period of 7 seconds and then kept at the corner for 3 seconds. This movement was repeated for 5 min. The time spent following the experimenter (S.O.)’s hand within a distance of approximately 3 cm was measured manually. In addition, the number of 50-kHz calls during the following behavior was recorded and counted. The experiment and analysis were conducted by experimenters (S.O. and Y.T.) blind to treatments of the animals.

#### Conditional place preference (CPP) test

The reward value of stroking stimuli was analyzed by a CPP test. The test apparatus consisted of a black box (W40 × D30 × H30 cm), a white box (W40 × D30 × H30 cm) and a boundary wall (30 × 30 cm) with a door hole (8 × 8 cm) through which rats could enter either box. Both boxes had ceilings in which video cameras were installed. The black box had black walls with a meshed floor, and its luminance was 10 lux. The white box had white walls with a grid floor, the grids being arranged in parallel with the boundary wall between the black and white boxes, and there was no illumination. On day 1, each rat was placed in the white box and habituated to the CPP test box for 10 min with the boundary door open so that the rat could enter either box. On day 2, each rat was placed in the black box with the door open for 10 min and then the time spent staying in each box was measured automatically by using software for behavior analysis (ImageJ LD1, O’Hara and Co.), which was produced on the basis of ImageJ program (National Institutes of Health, U.S.A.), to identify initial preference (pre-conditioning). The box in which each rat stayed for a longer time was defined as the initially preferred box (I.P. box) and the other box was defined as the initially non-preferred box (I.N.P. box). Locomotion distance in each box was also measured. Conditioning was performed from day 3 to day 8 with the door connecting the two boxes being closed. On day 3, rats were introduced into the I.P. box and kept there for 5 min. On day 4, rats were placed in the I.N.P. box and given stroking stimuli by the experimenter (S.O.) for 5 min. The set of 2-day conditioning procedures was repeated another 2 times from day 5 until day 8. On day 9, rats were placed in the I.P. box with the door open and the time spent in each box and distance of movements in each box were measured by using an analyzing program. In addition, [(time spent staying in the I.N.P. box during the post-conditioning)/(time spent staying in the I.N.P. box during the pre-conditioning)], [(locomotion distance in the I.N.P. box during the post-conditioning)/(locomotion distance in the I.N.P. box during the pre-conditioning)], and [(locomotion distance in the I.P. box during the post-conditioning)/(locomotion distance in the I.P. box during the pre-conditioning)] were used as preference ratios and were compared among the four groups.

### Immunohistochemical detection of c-Fos protein in oxytocin-immunoreactive (-ir) neurons

The rats in each group were assigned to one of two conditions: a stroking condition and a non-touch control condition. In the stroking condition, rats received stroking stimuli for 5 min and then they were placed back in their individual home cages. In the non-touch condition, rats were kept in their individual home cages. Ninety minutes after termination of stroking stimuli, the animals were anesthetized with Avertin (tribromoethanol; 200 mg/kg, i.p.) and perfused transcardially with heparinized saline (20 U/mL) followed by 4% paraformaldehyde in 0.1 M phosphate buffer (pH 7.4) for 15 min. The brains were immediately removed from the skulls, post-fixed in 4% paraformaldehyde overnight, placed in 30% sucrose in 0.1 M phosphate buffer until they sank, and frozen in dry ice. The hypothalamic part of each frozen brain was sectioned coronally at 30 µm and processed for immunochemical detection of c-Fos protein and oxytocin as described previously^29^. Sections containing the dorsal zone of the medial parvicellular part of the caudal PVN (3 sections, from 1.92 mm to 2.16 mm posterior to the bregma), rostral PVN (6 sections, from 1.2 mm to 1.80 mm posterior to the bregma), supraoptic nuclei (SON, 7 sections, from 0. 6 mm to 1.32 mm posterior to the bregma) or BNST (4 sections, from 0.72 mm to 1.08 mm posterior to the bregma) were examined at an interval of 120 µm in each rat. The sum of the numbers of c-Fos-ir neurons, oxytocin-ir neurons, and double-positive neurons were counted in each brain region, the boundary of which was determined according to a brain atlas^42^. The percentage of oxytocin-ir neurons expressing c-Fos protein was determined.

### Statistical analysis

**Figure 2:**
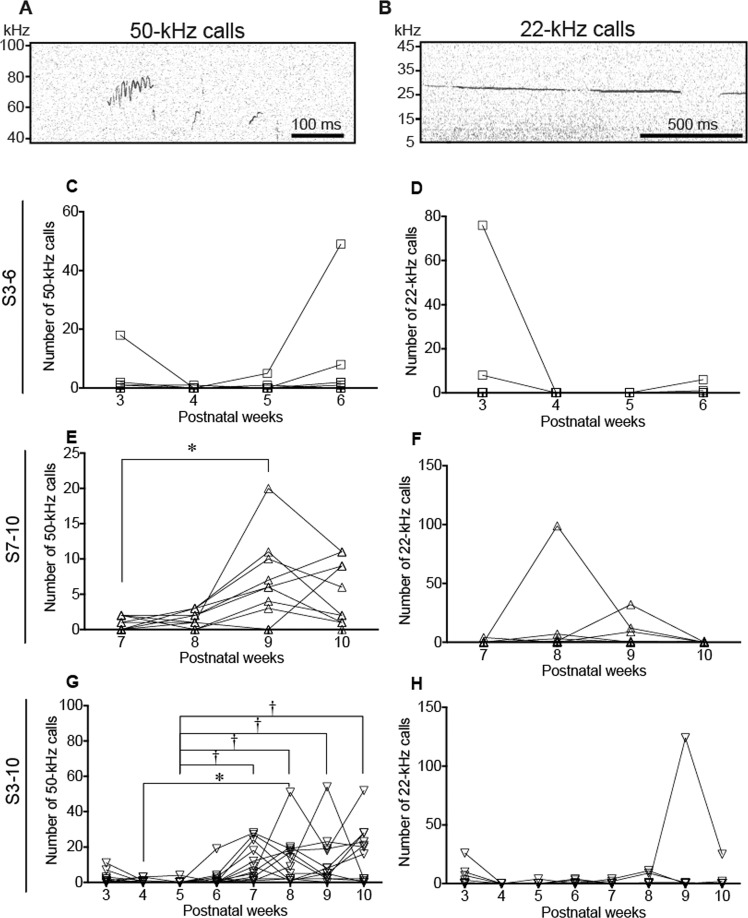
Ultrasonic vocalizations (USVs) during stroking stimuli. (**A,B**) Examples of spectrograms of 50-kHz calls (**A**) and 22-kHz calls (**B**). Time courses of the numbers of vocalizations during stroking in the S3–6 group (C, 50-kHz; D, 22-kHz), S7–10 group (E, 50-kHz; F, 22-kHz), and S3–10 group (G, 50-kHz; H, 22-kHz). In the S3–6 group, the number of 50-kHz calls did not significantly change. In the S7–10 group, the number of 50-kHz calls at the age of 9 weeks was significantly larger than that at the age of 7 weeks. In the S3–10 group, the numbers of 50-kHz calls at 7, 8, 9, and 10 weeks of age were significantly increased compared to the number at the age of 5 weeks. In addition, the number of 50-kHz calls at 8 weeks of age was significantly larger than that at 4 weeks of age. The numbers of 22-kHz calls during stroking did not significantly change in any of the three groups. ^†^*P* < 0.01; *P < 0.05, post-hoc Dunn’s test.

**Figure 3:**
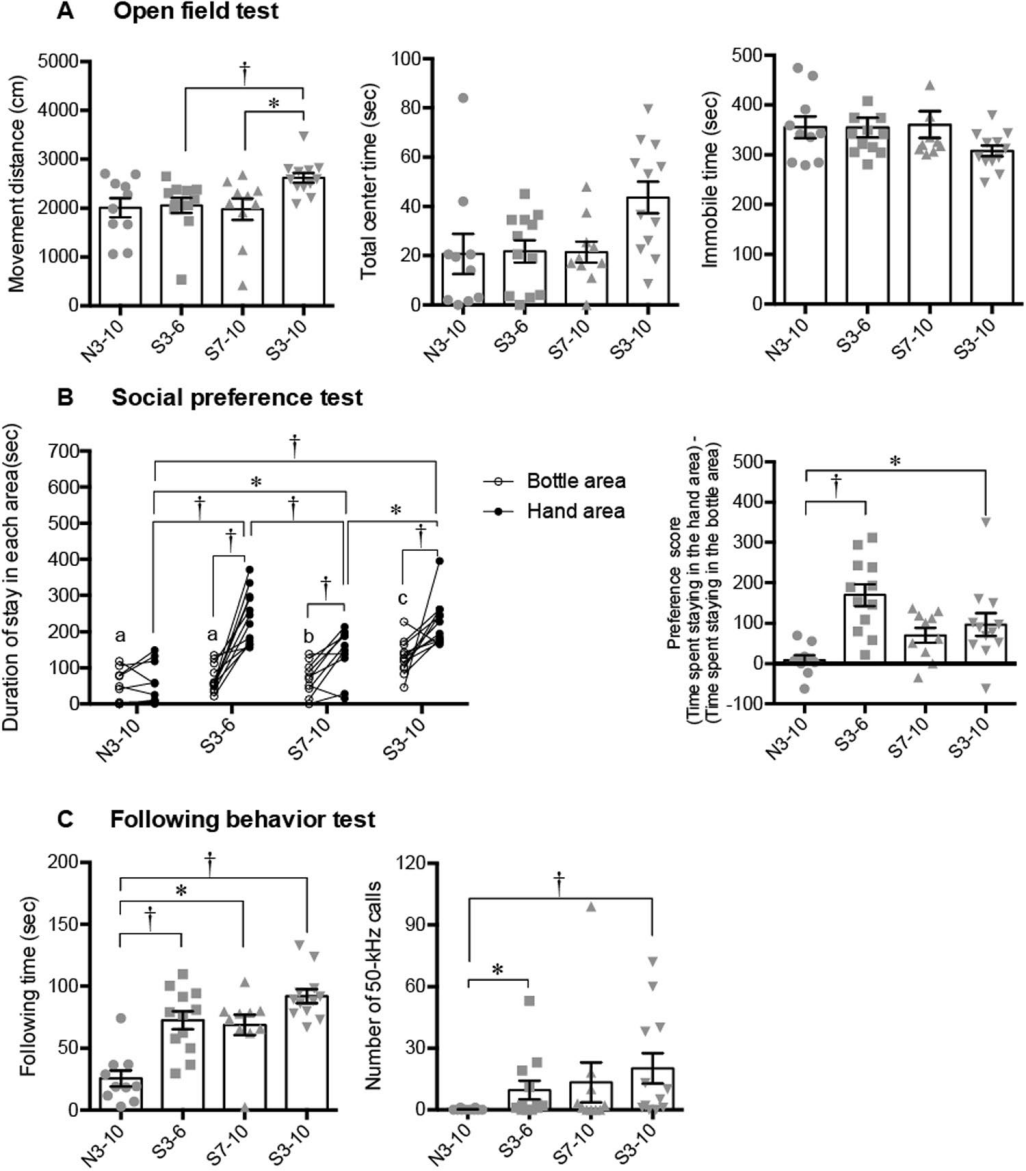
Results of the open field test, social preference test, and following behavior test. (**A**) Total distance of movements (left panel), total time spent staying in the center area (middle panel), and total time spent immobile (right panel) in an open field test. There were no significant differences in the total center time and immobile time among the groups. (**B**) Difference between durations of staying in the hand area and the bottle area in a social preference test. The time spent staying in the hand area was significantly longer than that in the bottle area in the S3–6, S7–10 and S3–10 groups. Preference scores [(time spent staying in the hand area) – (time spent staying in the bottle area)] in the S3–6 and S3–10 groups were significantly higher than that in N3–10 group. (**C**) Durations of following behavior and number of 50-kHz calls during a following behavior test. Durations of following behavior (left panel) were significantly longer in the S3–6, S7–10, and S3–10 groups than in the N3–10 group. The numbers of 50-kHz calls (right panel) were significantly larger in the S3–6 and S3–10 groups than in the N3–10 group. ^†^*P* < 0.01; *P < 0.05, a versus c, *P* < 0.01, b versus c, *P* < 0.05. Post-hoc Steel-Dwass test (A, right graph of B, and C) and repeated measures two-way ANOVA (group × area) followed by post-hoc Holm’s test (left graph of B) were performed. Error bars denote standard error of the mean.

**Figure 4:**
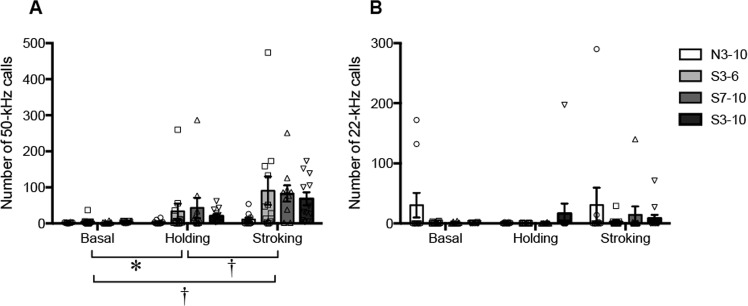
Numbers of 50-kHz (**A**) and 22-kHz (**B**) calls. The number of 50-kHz calls in the stroking condition was significantly larger than that in the basal or holding condition. The number of 22-kHz calls did not significantly change. ^†^*P* < 0.01; *P < 0.05. Repeated measures two-way ANOVA (group × condition) followed by post-hoc Holm’s test was performed. Error bars denote standard error of the mean.

**Figure 5:**
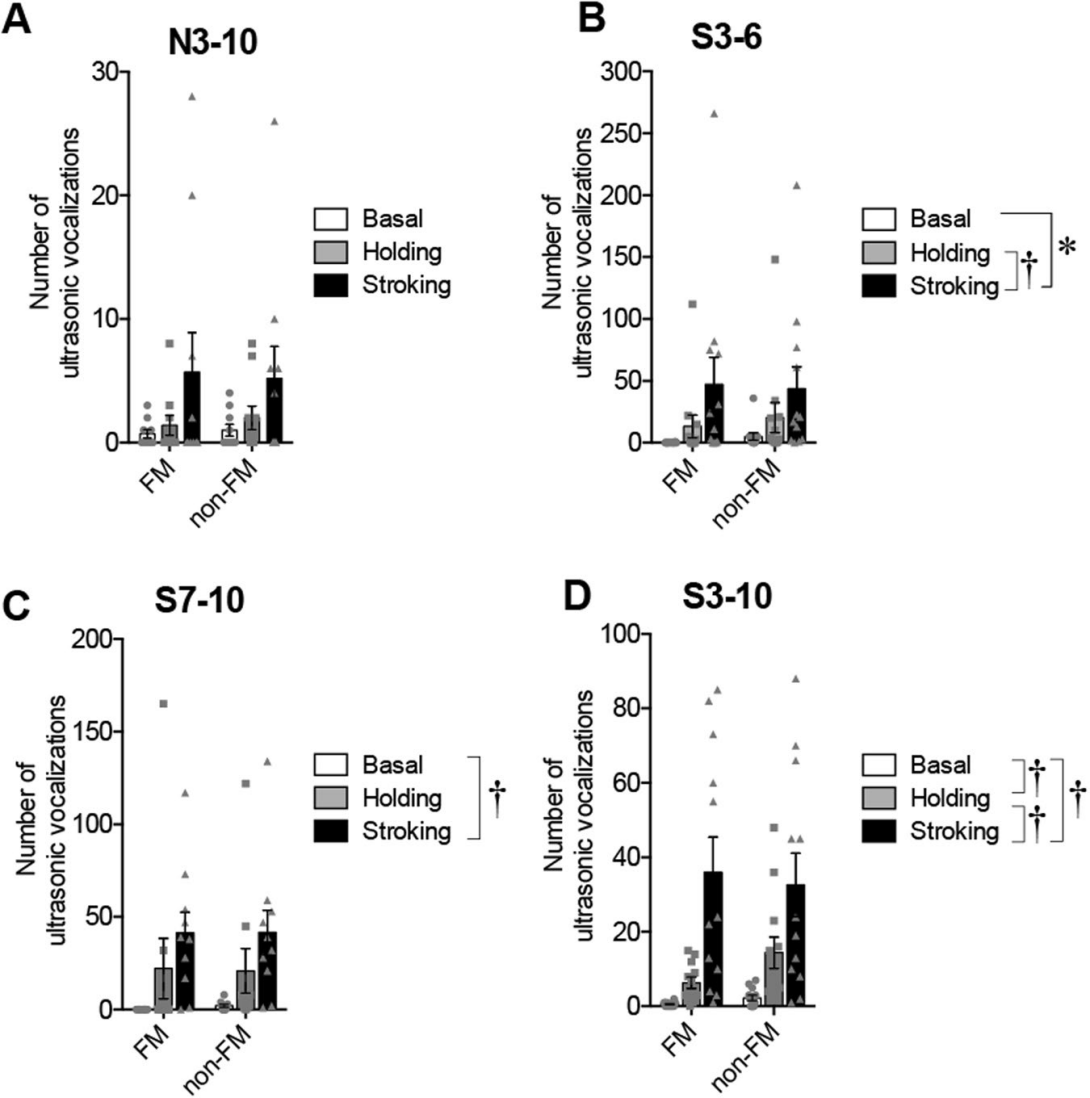
Numbers of FM and non-FM calls in the N3–10 (**A**), S3–6 (**B**), S7–10 (**C**), and S3–10 (**D**) groups. In the S3–6 group, the number of calls in the stroking condition was significantly larger than that in the basal or holding condition. In the S7–10 group, the number of calls in the stroking condition was significantly larger than that in the basal condition. In the S3–10 group, the number of calls in the stroking condition was significantly larger than that in the basal or holding condition. In addition, the number of calls in the holding condition was significantly larger than that in the basal condition. ^†^*P* < 0.01, *P < 0.05. A comparison among conditions was conducted by repeated measures two-way ANOVA (syllable pattern × condition) followed by post-hoc Holm’s test. Error bars denote standard error of the mean.

Statistical analyses were performed using JMP 13.0.0 software (SAS Institute, NC, USA), Prism 6 (GraphPad Software Inc., CA, USA), R and SPSS Statistics 22 (IBM, IL, USA). The numbers of USVs at 3, 4, 5, 6, 7, 8, 9, and 10 weeks in the three stroking groups were analyzed separately for within-group comparisons by using Friedman’s test followed by the post-hoc Dunn’s test (Fig. 2C–H). Data for the open field test (Fig. 3A), preference score of the social preference test (Fig. 3B right), following behavior test (Fig. 3C), and number of USVs during the following behavior test (Fig. 3C) were analyzed by the Kruskal-Wallis test followed by the post-hoc Steel-Dwass test. Times spent in the bottle and hand areas in the social preference test (Fig. 3B left) were analyzed by repeated measures two-way analysis of variance (ANOVA) (group × area) followed by post-hoc Holm’s test. Data for the numbers of 50-kHz and 22-kHz calls in basal, holding, and stroking conditions (Fig. 4) were also analyzed by repeated measures two-way ANOVA (group × condition) followed by post-hoc Holm’s test. The numbers of vocalizations in FM and non-FM and those of each syllable (Fig. 5 and supplementary Fig. 1) were analyzed by repeated measures two-way ANOVA (syllable pattern × condition) followed by post-hoc Holm’s test.

**Figure 6:**
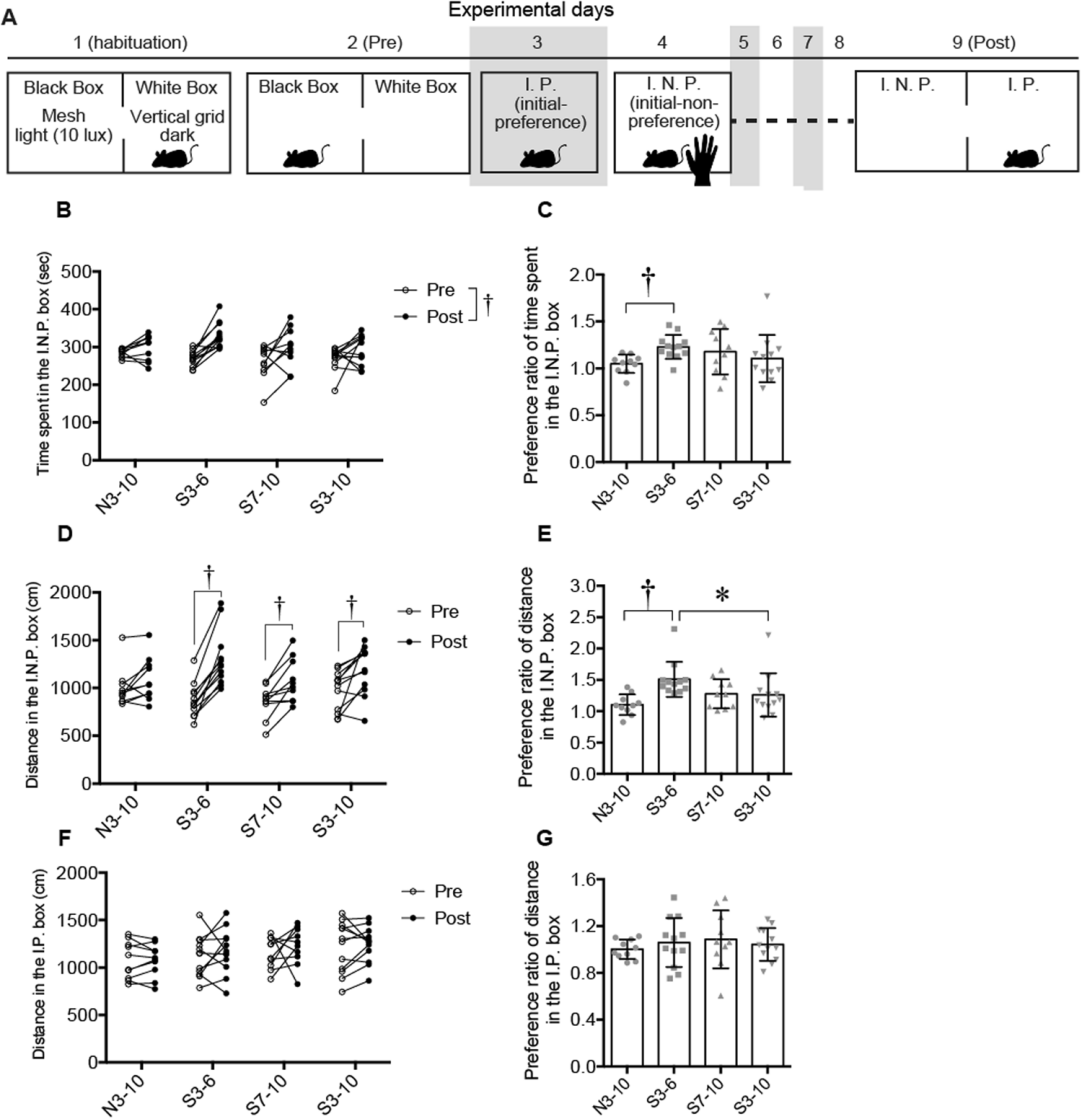
Results of the conditional place preference test. (**A**) Time course of experimental procedures for the conditioned place preference test. (**B**) Times spent staying in an initial non-preference (I.N.P.) box before (pre) and after (post) conditioning. Time spent staying in an I.N.P. box where rats received stroking stimuli was significantly longer after conditioning than that before conditioning. (**C**) Preference ratio of time [(time spent staying in the I.N.P. box during the post-conditioning)/(time spent staying in the I.N.P. box during the pre-conditioning)] was significantly higher in the S3–6 group than in the N3–10 group. (**D**) Distance of movements in the I.N.P. box before conditioning (pre) and that after conditioning (post). In the S3–6, S7–10, and S3–10 groups, the distance of movements in the I.N.P. box was significantly increased after conditioning compared to that before conditioning. (**E**) Preference ratio of locomotion distance [(locomotion distance in the I.N.P. box during the post-conditioning)/(locomotion distance in the I.N.P. box during the pre-conditioning)] was significantly higher in the S3–6 group than in the N3–10 and S3–10 groups (**F**) Distance of movements in the initial preference (I.P.) box before conditioning (pre) and that after conditioning (post). No significant differences were found. (**G**) Preference ratio of locomotion distance in the I.P. box. Preference ratio of locomotion distance in the I.P. box also did not significantly change in any of the groups. ^†^*P* < 0.01; *P < 0.05. Repeated measures two-way ANOVA (group × timing) followed by post-hoc Holm’s test (**B**, **D**, and **F**), and the Kruskal-Wallis test followed by a post-hoc Steel-Dwass test (**C**, **E**, and **G**) were used. Error bars denote standard error of the mean.

**Figure 7:**
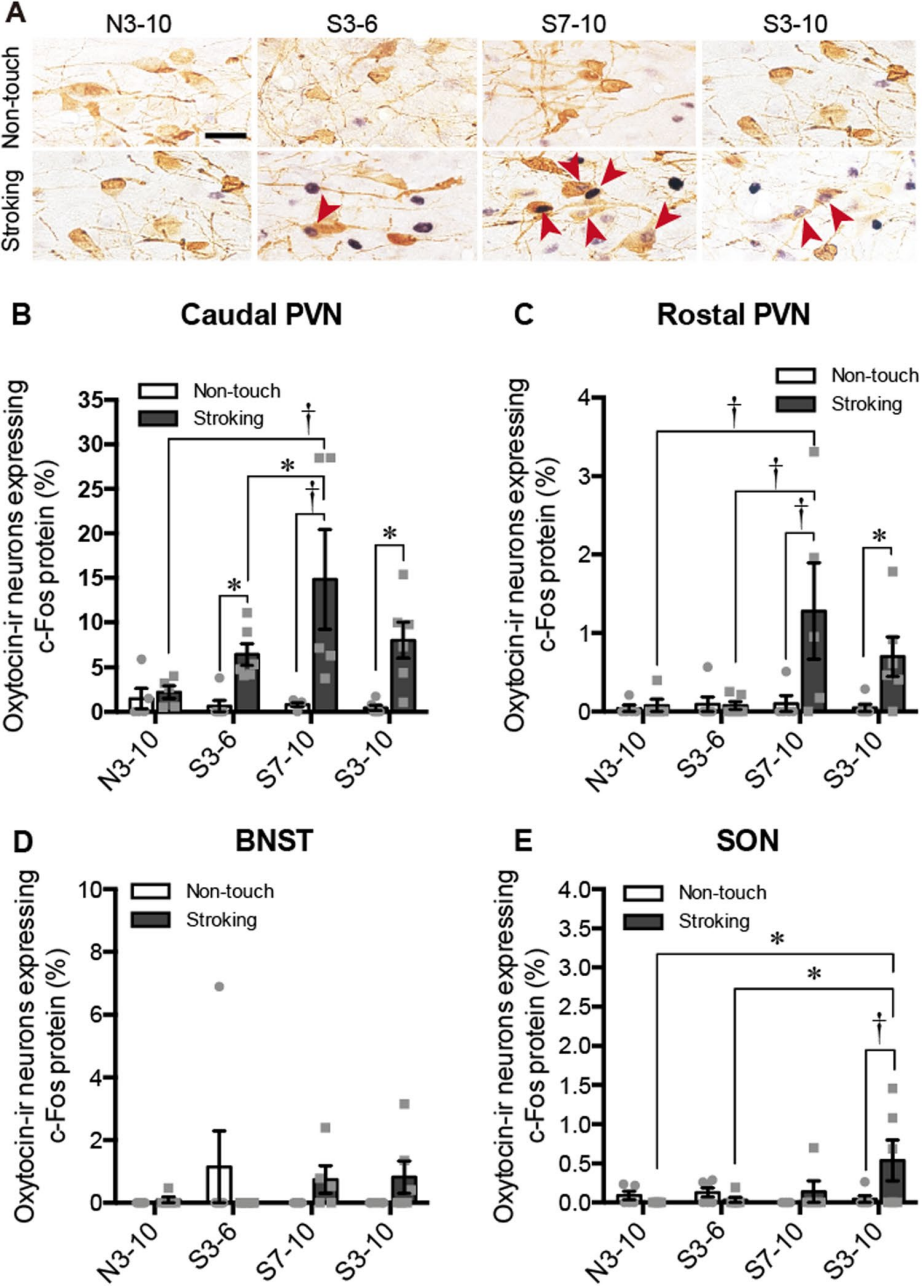
Results of immunohistochemical detection. (**A**) Photographs showing c-Fos immunoreactivity (black nuclear profiles) in oxytocin-ir neurons (brown cell body profiles). Expression of c-Fos protein in oxytocin-immunoreactive (-ir) neurons of the caudal hypothalamic paraventricular nucleus (PVN) following a non-touch condition or stroking condition in the N3–10, S3–6, S7–10, and S3–10 groups was examined. (**B–E**) Percentages of oxytocin-ir neurons expressing c-Fos protein in the caudal PVN (**B**), rostral PVN (**C**), bed nucleus of the stria terminalis (BNST) (**D**), and supraoptic nucleus (SON) (**E**). In the caudal PVN, stroking stimuli increased expression of c-Fos protein in oxytocin-ir cells in the S3–6, S7–10 and S3–10 groups. In the rostral PVN, stroking stimuli significantly increased the percentage of oxytocin-ir cells expressing c-Fos protein in the S7–10 and S3–10 groups compared to that in the non-touch condition. The percentage of oxytocin-ir cells expressing c-Fos protein after stroking stimuli in the S7–10 group was significantly higher than that in the N3–10 or S3–6 group in the caudal PVN and rostra PVN. In the BNST, no significant differences in the percentage of oxytocin-ir cells expressing c-Fos protein were found. In the SON, stroking stimuli significantly increased the percentage of oxytocin-ir cells expressing c-Fos protein in the S3–10 group, and the percentage of those cells in the S3–10 group was significantly higher than that in the N3–10 or S3–6 group. ^†^*P* < 0.01, *P < 0.05. Two-way factorial ANOVA (group × stimuli) followed by post-hoc Holm’s test was performed. Error bars denote standard error of the mean. The red arrowheads indicate double-labeled neurons. Scale bar = 40 μm.

Data for CPP (Fig. 6) were analyzed by repeated measures two-way ANOVA (group × timing) followed by post-hoc Holm’s test or the Kruskal-Wallis test followed by the post-hoc Steel-Dwass test. Results of immunohistochemical experiments (Fig. 7 and supplementary Fig. 2) were analyzed by two-way factorial ANOVA (group × stimuli) followed by post-hoc Holm’s test. In post hoc multiple comparisons, every possible comparison was conducted and *P* values were adjusted by Holm’s method. Correlations between percentages of oxytocin-ir neurons expressing c-Fos protein in the caudal PVN and behavioral data (results of the open field test, social preference test and following behavior test, results for 50-kHz calls during the stroking condition, and results of the CPP test) and correlations between the number of 50-kHz calls during the stroking condition and other behavioral data were analyzed by Spearman’s correlation test. Data of all 4 groups were collapsed into a single analysis for correlation analysis. *P* < 0.05 was considered statistically significant.

## Results

### Developmental changes of ultrasonic vocalizations

In the developmental period, the number of 50-kHz calls rats emitted during stroking stimuli was small and did not significantly increase between the ages of 3 weeks and 6 weeks (Fig. 2C) in the S3–6 group. In the S7–10 group, the number of 50-kHz calls during stroking stimuli increased and the number of 50-kHz calls at the age of 9 weeks was significantly increased compared to that at the age of 7 weeks (Fig. 2E, *P* = 0.0289, Friedman’s test, *P* = 0.0437, post-hoc Dunn’s test). In the S3–10 group, the number of 50-kHz calls gradually increased and the numbers of calls at 7, 8, 9, and 10 weeks of age were significantly increased compared to the number of calls at 5 weeks of age (Fig. 2G, *P* < 0.0001, Friedman’s test, 5 weeks versus 7 weeks, *P* = 0.0018; 5 weeks versus 8 weeks, *P* = 0.0013; 5 weeks versus 9 weeks, *P* = 0.0036; 5 weeks versus 10 weeks, *P* = 0.0021, post-hoc Dunn’s test). In addition, the number of 50-kHz calls at 8 weeks of age was significantly larger than that at 4 weeks of age (Fig. 2G, *P* < 0.0001, Friedman’s test, *P* = 0.0434, post-hoc Dunn’s test).

The number of 22-kHz calls during stroking did not significantly change in any of the three groups (Fig. 2D,F,H).

### Open field test

Total distances of locomotion in the S3–6 group, S7–10 group and S3–10 group were not significantly different from the total distance in the N3–10 group, although the total distance of locomotion in the S3–10 group was significantly longer than the total distances in the S3–6 and S7–10 groups (Fig. 3A left, *χ*^2^ = 12.3292, *df* = 3, *P* = 0.0063, Kruskal-Wallis test; S3–10 group versus S3–6 group, *P* = 0.0074; S3–10 group versus S7–10 group, *P* = 0.038, post-hoc Steel-Dwass test). There were no significant differences in total center times and immobile times among the groups (Fig. 3A, middle and right).

### Social preference test

Repeated measures two-way ANOVA revealed a significant effect of area, a significant effect of group [area, F (1, 40) = 53.8092, *P* < 0.0001; group, F (3, 40) = 20.1714, *P* < 0.0001], and a significant interaction of the two [F (3, 40) = 8.0318, *P* = 0.0003]. The time spent staying in the hand area was significantly longer than that in the bottle area in the S3–6, S7–10 and S3–10 groups (Fig. 3B left, S3–6, *P* = 0.0001; S7–10, P = 0.0044; S3–10, *P* = 0.0056, post-hoc Holm’s test). The time spent staying in the hand area in all three stroking groups was significantly longer than that in the N3–10 group (N3–10 versus S3–6, *P* < 0.0001; N3–10 versus S7–10, *P* = 0.0147; N3–10 versus S3–10, *P* < 0.0001; S3–6 versus S7–10, *P* = 0.0055; S7–10 versus S3–10, *P* = 0.0147, post-hoc Holm’s test), while the time spent staying in the bottle area in the S3–10 group was significantly longer than that in other groups (N3–10 versus S3–10, *P* = 0.0006; S3–10 versus S3–6, *P* = 0.0094; S3–10 versus S7–10, *P* = 0.0144, post-hoc Holm’s test).

Preference scores [(time spent staying in the hand area) – (time spent staying in the bottle area)] in the S3–6 and S3–10 groups were significantly higher than the preference score in the N3–10 group (Fig. 3B right, *χ*^2^ = 18.5844, *df* = 3, *P* = 0.0003, Kruskal-Wallis test; N3–10 group versus S3–6 group, *P* = 0.0011; N3–10 versus S3–10 group, *P* = 0.0215, Steel-Dwass test).

### Following behavior test

The periods of time spent for following behavior toward the hand of the experimenter (S.O.) were significantly longer in the S3–6, S7–10, and S3–10 groups than in the N3–10 group (Fig. 3C left, *χ*^2^ = 21.1022, *df* = 3, *P* = 0.0001, Kruskal-Wallis test; N3–10 group versus S3–6 group, *P* = 0.0038; N3–10 group versus S7–10 group, *P* = 0.0366; N3–10 group versus S3–10 group, *P* = 0.0009, post-hoc Steel-Dwass test).

The numbers of 50-kHz calls during the following behavior test in the S3–6 and S3–10 groups were significantly larger than the number in the N3–10 group (Fig. 3C right, *χ*^2^ = 11.8594, *df* = 3, *P* = 0.0079, Kruskal-Wallis test; N3–10 group versus S3–6 group, *P* = 0.0268; N3–10 group versus S3–10 group, *P* = 0.0086, post-hoc Steel-Dwass test).

### Ultrasonic vocalizations in basal, holding and stroking conditions

#### 50-kHz calls and 22-kHz calls

In the number of 50-kHz calls (Fig. 4A), there was no significant effect of group [F (3, 40) = 1.6853, *P* = 0.1855], but there was a significant effect of condition [F (1.59, 63.48) = 16.272, *P* < 0.0001] and there was no significant interaction [F (4.76, 63.48) = 1.3693, *P* = 0.2494, repeated measures two-way ANOVA]. The number of 50-kHz calls was significantly larger in the holding condition than in the basal condition (*P* = 0.0167, post-hoc Holm’s test), and it was significantly larger in the stroking condition than in the basal or holding condition (stroking versus basal condition, *P* = 0.0001; stoking versus holding condition, *P* = 0.0006, post-hoc Holm’s test), suggesting that 50-kHz calls were induced by stroking stimuli.

On the other hand, the numbers of 22-kHz calls were not significantly different in the basal, holding, and stroking conditions. In the numbers of 22-kHz calls (Fig. 4B), there was no significant effect of group [F (3, 40) = 1.0464, *P* = 0.3826] or condition [F (1.52, 60.94) = 0.9347, *P* = 0.3755] and there was no significant interaction [F (4.57, 60.94) = 1.0906, *P* = 0.3727, repeated measures two-way ANOVA].

#### Syllable patterns of ultrasonic vocalizations

In the numbers of total FM and total non-FM calls in the N3–10 group (Fig. 5A), there was no significant effect of syllable pattern [F (1, 18) = 0.0067, *P* = 0.9358], but there was a significant effect of condition [F (1.21, 21.73) = 4.5066, *P* = 0.0391] and there was no significant interaction [F (1.21, 21.73) = 0.0608, *P* = 0.8517, repeated measures two-way ANOVA]. Post hoc analysis revealed no significant difference in the number of calls among conditions.

In the number of vocalizations of each syllable subtype for the N3–10 group (Supplementary Fig. 1A), there were significant effects of syllable subtype [F (11, 108) = 2.5750, *P* = 0.0061] and condition [F (1.2, 129.51) = 9.6727, *P* = 0.0013], but there was no significant interaction [F (13.19, 129.51) = 1.5000, *P* = 0.1245, repeated measures two-way ANOVA]. The number of calls in the stroking condition was significantly larger than that in the basal or holding condition (stroking versus basal condition, *P* = 0.0044; stroking versus holding condition, *P* = 0.0049, post-hoc Holm’s test). Post-hoc Holm’s test revealed no significant difference in the number of calls among syllable subtypes (see supplementary Fig. 1A).

In the numbers of total FM and total non-FM calls in the S3–6 group (Fig. 5B), there was no significant effect of syllable pattern [F (1, 22) = 0.0416, *P* = 0.8402], but there was a significant effect of condition [F (1.14, 25.07) = 8.6723, *P* = 0.0053] and there was no significant interaction [F (1.14, 25.07) = 0.1400, *P* = 0.7445, repeated measures two-way ANOVA]. The number of calls in the stroking condition was significantly larger than that in the basal or holding condition (stroking versus basal condition, *P* = 0.0135; stroking versus holding condition, *P* = 0.0042, post-hoc Holm’s test).

In the number of vocalizations of each syllable subtype for the S3–6 group (Supplementary Fig. 1B), there were significant effects of syllable subtype [F (11, 132) = 3.1737, *P* = 0.0008] and condition [F (1.3, 171.14) = 23.3754, *P* < 0.0001] and there was a significant interaction [F (14.26, 171.14) = 2.3816, *P* = 0.0045, repeated measures two-way ANOVA]. Post-hoc Holm’s test showed significant differences in the number of calls among syllable subtypes and conditions (see supplementary Fig. 1B).

In the numbers of total FM and total non-FM calls in the S7–10 group (Fig. 5C), there was no significant effect of syllable pattern [F (1, 18) = 0.0008, *P* = 0.9771] but there was a significant effect of condition [F (2, 36) = 9.2524, *P* = 0.0006] and there was no significant interaction [F (2, 36) = 0.0154, *P* = 0.9847, repeated measures two-way ANOVA]. The number of calls in the stroking condition was significantly larger than that in the basal condition (*P* = 0.0003, post-hoc Holm’s test).

In the number of vocalizations of each syllable subtype for the S7–10 group (Supplementary Fig. 1C), there were significant effects of syllable subtype [F (11, 108) = 5.6758, *P* < 0.0001] and condition [F (1.91, 206.28) = 23.4432, *P* < 0.0001], and there was a significant interaction [F (21.01, 206.28) = 3.1075, *P* < 0.0001, repeated measures two-way ANOVA]. Post-hoc Holm’s test showed significant differences in the number of calls among syllable subtypes and conditions (see supplementary Fig. 1C).

In the numbers of total FM and total non-FM calls in the S3–10 group (Fig. 5D), there was no significant effect of syllable pattern [F (1, 22) = 0.1722, *P* = 0.6822] but there was a significant effect of condition [F (1.19, 26.19) = 22.1925, *P* < 0.0001] and there was no significant interaction [F (1.19, 26.19) = 0.6379, *P* = 0.4580, repeated measures two-way ANOVA]. The number of calls in the stroking condition was significantly larger than that in the basal (*P* = 0.0001) or holding condition (*P* = 0.0009, post-hoc Holm’s test). In addition, the number of calls in the holding condition was significantly larger than that in the basal condition (*P* = 0.0009, post-hoc Holm’s test).

In the number of vocalizations of each syllable subtype for the S3–10 group (Supplementary Fig. 1D), there were significant effects of syllable subtype [F (11, 132) = 10.9240, *P* < 0.0001] and condition [F (1.25, 164.62) = 50.5745, *P* < 0.0001] and there was a significant interaction [F (13.72, 164.62) = 6.6464, *P* < 0.0001, repeated measures two-way ANOVA]. Post-hoc Holm’s test showed significant differences in the number of calls among syllable subtypes and conditions (see supplementary Fig. 1D).

### Conditional place preference (CPP) test

In the time spent staying in the I.N.P. box where rats received stroking stimuli (Fig. 6B), there was no significant effect of group [F (3, 40) = 1.4404, *P* = 0.2453] but there was a significant effect of timing [F (1, 40) = 24.4405, *P* < 0.0001] and there was no significant interaction [F (3, 40) = 2.2999, *P* = 0.0919, repeated measures two-way ANOVA]. The time spent staying in a box where rats received stroking stimuli was significantly longer after conditioning with stroking stimuli than that before conditioning (pre versus post, *P* < 0.001). The preference ratio of time [(time spent staying in the I.N.P. box during the post-conditioning)/(time spent staying in the I.N.P. box during the pre-conditioning)] was significantly higher in the S3–6 group than in the N3–10 group (Fig. 6C, *χ*^2^ = 8.8665, *df* = 3, *P* = 0.0311, Kruskal-Wallis test, *P* = 0.0094, post-hoc Steel-Dwass test).

In the distances of locomotion in the box where rats received stroking stimuli (Fig. 6D), there was no significant effect of group [F (3, 40) = 0.8016, *P* = 0.5003] but there was a significant effect of timing [F (1, 40) = 68.1068, *P* < 0.0001] and there was a significant interaction [F (3, 40) = 5.6745, *P* = 0.0025, repeated measures two-way ANOVA]. Distances of locomotion in the box where rats received stroking stimuli were significantly increased compared to those before receiving conditioning in the S3–6, S7–10, and S3–10 groups (pre versus post, S3–6, *P* < 0.0001; S7–10, *P* = 0.0024; S3–10, *P* = 0.0075). The preference ratio of locomotion distance [(locomotion distance in the I.N.P. box during the post-conditioning)/(locomotion distance in the I.N.P. box during the pre-conditioning)] in the S3–6 group was significantly higher than the ratios in the N3–10 and S3–10 groups (Fig. 6E, *χ*^2^ = 14.5939, *df* = 3, *P* = 0.0022, Kruskal-Wallis test, S3–6 group versus N3–10 group, *P* = 0.0024; S3–6 group versus S3–10 group, *P* = 0.031, post-hoc Steel-Dwass test).

On the other hand, in the locomotion activity in the I.P. box where rats did not receive stroking stimuli (Fig. 6F), there was no significant effect of group [F (3, 40) = 1.3501, *P* = 0.2719] or timing [F (1, 40) = 1.3675, *P* = 0.2492] and there was no significant interaction [F (3, 40) = 0.2589, *P* = 0.8545, repeated measures two-way ANOVA]. The preference ratio of locomotion distance in the I.P. box also did not significantly change in any of the groups (Fig. 6G).

### Expression of c-Fos protein in oxytocin-immunoreactive (-ir) neurons

In the percentages of oxytocin-ir neurons expressing c-Fos protein in the caudal PVN (Fig. 7B), there was no significant effect of group [F (3, 36) = 2.7195, *P* = 0.0588] but there was a significant effect of stimuli [F (1, 36) = 22.9003, *P* < 0.0001] and there was a significant interaction [F (3, 36) = 3.2390, *P* = 0.0333, two-way ANOVA]. The percentage of oxytocin-ir neurons expressing c-Fos protein was significantly higher after stroking in the S3–6, S7–10, and S3–10 groups but not in the N3–10 group than that in non-stroking control rats (non-touch vs stroking, S3–6, *P* = 0.0466; S7–10, *P* = 0.0001; S3–10, *P* = 0.0104). The percentage of oxytocin-ir cells expressing c-Fos protein following the stroking stimuli was significantly higher in the S7–10 group than in the N3–10 or S3–6 group (Fig. 7B, S7–10 versus N3–10, *P* = 0.0013; S7–10 versus S3–6, *P* = 0.0338, post-hoc Holm’s test).

In the percentages of oxytocin-ir neurons expressing c-Fos protein in the rostral PVN (Fig. 7C), there were significant effects of group [F (3, 36) = 3.1916, *P* = 0.0350] and stimuli [F (1, 36) = 8.2396, *P* = 0.0068] and there was a significant interaction [F (3, 36) = 2.9704, *P* = 0.0446, two-way ANOVA]. The percentages of oxytocin-ir neurons expressing c-Fos protein were increased after stroking stimuli in the S7–10 and S3–10 groups compared with that in non-stroking control rats (non-touch versus stroking, S7–10, *P* = 0.0013; S3–10, *P* = 0.0413). The percentage of oxytocin-ir cells expressing c-Fos protein following the stroking stimuli was significantly higher in the S7–10 group than in the N3–10 or S3–6 group (S7–10 versus N3–10, *P* = 0.0052; S7–10 versus S3–6, *P* = 0.004, post-hoc Holm’s test).

In the percentages of oxytocin-ir cells expressing c-Fos protein in the bed nucleus of the stria terminalis (BNST) (Fig. 7D), there was no significant effect of group [F (3, 36) = 0.3640, *P* = 0.7793] or stimuli [F (1, 36) = 0.1299, *P* = 0.7279] and there was no significant interaction [F (3, 36) = 1.6922, *P* = 0.1860, two-way ANOVA].

In the percentages of oxytocin-ir cells expressing c-Fos protein in the supraoptic nucleus (SON) (Fig. 7E), there was no significant effect of group [F (3, 36) = 2.0091, *P* = 0.1300] or stimuli [F (1, 36) = 1.8274, *P* = 0.1849] but there was a significant interaction [F (3, 36) = 3.0090, *P* = 0.0428, two-way ANOVA]. In the percentage of oxytocin-ir cells expressing c-Fos protein after stroking stimuli was higher than that in the non-touch group in the S3–10 group (stroking versus non-touch in the S3–10 group, *P* = 0.0034). The percentage of oxytocin-ir cells expressing c-Fos protein following the stroking stimuli was significantly higher in the S3–10 group than in the N3–10 or S3–6 group (Fig. 7E, S3–10 versus N3–10, *P* = 0.0147; S3–10 versus S3–6, *P* = 0.0147, post-hoc Holm’s test).

The numbers of oxytocin-ir cells in the caudal PVN, rostral PVN, BNST, and SON were not significantly different among the four groups. In the numbers of oxytocin-ir cells in the caudal PVN, rostral PVN, BNST, and SON, there were no significant effect of group, no significant effect of stimuli, and no significant interaction [Supplementary Fig. 2, caudal PVN, F (3, 36) = 0.4363, *P* = 0.7284, no significant effect of group, F (1, 36) = 0.2820, *P* = 0.5986, no significant effect of stimuli, F (3, 36) = 0.4479, *P* = 0.7203, no significant interaction; rostral PVN, F (3, 36) = 0.6228, *P* = 0.6049, no significant effect of group, F (1, 36) = 0.0252, *P* = 0.8749, no significant effect of stimuli, F (3, 36) = 1.1921, *P* = 0.3265, no significant interaction; BNST, F (3, 36) = 2.4283, *P* = 0.0812, no significant effect of group, F (1, 36) = 3.7307, *P* = 0.0613, no significant effect of stimuli, F (3, 36) = 0.6831, *P* = 0.5682, no significant interaction; SON, F (3, 36) = 0.8253, *P* = 0.4886, no significant effect of group, F (1, 36) = 0.1597, *P* = 0.6918, no significant effect of stimuli F (3, 36) = 0.4561, *P* = 0.7146, no significant interaction, two-way ANOVA].

The numbers of non-oxytocin-ir neurons expressing immunoreactivity of c-Fos protein were significantly increased after stroking stimuli in the caudal PVN, rostral PVN, and BNST but not in the SON. In the numbers of non-oxytocin-ir neurons expressing immunoreactivity of c-Fos protein in the caudal PVN, rostral PVN, and BNST but not in the SON, there were no significant effect of group, but significant effect of stimuli and no significant interaction [Supplementary Fig. 2, caudal PVN, F (3, 36) = 0.3287, *P* = 0.8046, no significant effect of group, F (1, 36) = 23.2825, *P* < 0.0001, significant effect of stimuli, F (3, 36) = 0.3759, *P* = 0.7709, no significant interaction; rostral PVN, F (3, 36) = 0.0435, *P* = 0.9877, no significant effect of groups, F (1, 36) = 22.7736, *P* < 0.0001, significant effects of stimuli, F (3, 36) = 0.8750, *P* = 0.4631, no significant interaction; BNST, F (3, 36) = 1.2551, *P* = 0.3043, no significant effect of group, F (1, 36) = 42.8085, *P* < 0.0001, significant effect of stimuli, F (3, 36) = 0.200, *P* = 0.8957, no significant interaction; SON, F (3, 36) = 1.5361, *P* = 0.2218, no significant effect of group, F (1, 36) = 0.1780, *P* = 0.6756, no significant effect of stimuli, F (3, 36) = 1.2771, *P* = 0.2969, no significant interaction, two-way ANOVA].

### Correlation analysis

Significant positive correlations were found between the percentage of oxytocin-ir neurons expressing c-Fos protein in the caudal PVN and the number of 50-kHz calls during the stroking condition and between the percentage of oxytocin-ir neurons expressing c-Fos protein in the caudal PVN and the preference ratio of locomotion distance in the I.N.P. box (Supplementary Table 1).

The number of 50-kHz calls during the stroking condition showed significant positive correlations with movement distance and total center time in the open field test, with the duration of following behavior and number of 50-kHz calls during the following behavior test, and with the preference ratio of time spent staying in the I.N.P. box (Supplementary Table 2). In addition, a significant negative correlation was found between the number of 50-kHz calls during the stroking condition and the immobile time in the open field test (Supplementary Table 2).

## Discussion

In the present study, post-weaning stroking stimuli for more than 4 weeks facilitated emission of 50-kHz calls, particularly FM-calls, which have been shown to be associated with positive emotional states^20^, and induced following behavior toward the experimenter’s hand. The findings suggest that repeated stroking stimuli for more than 4 weeks induced positive emotion in rats in response to stroking stimuli. Post-weaning stroking stimuli between 3 and 6 weeks of age induced preference and following behavior toward the experimenter’s hand and increased reward values of stroking stimuli as indicated by the CPP test. All of the data suggest that post-weaning stroking during the early adolescent period facilitated the establishment of an interspecific affiliative relationship between rats and humans. The results showing that stroking stimuli activated oxytocin neurons in the hypothalamus are consistent with the view that oxytocin plays an important role for an affiliative relationship between not only conspecifics such as mother-infant or male and female pairs but also interspecific animals including humans and rats.

During the developmental period of 3–6 weeks of age, the S3–10 group showed a small number of 50-kHz calls in response to stroking stimuli, while the number of 50-kHz calls gradually increased after 7 weeks of age. Previous studies showed that juvenile rats at the postnatal 35^th^ day emit a large number of 50-kHz calls during playing behavior^24^. Thus, it is likely that rats of postnatal 3–6 weeks of age had the ability to emit 50-kHz calls but did not emit 50-kHz calls in response to gentle stroking stimuli in the present study. The findings suggest that rats changed their emotional reaction toward stroking stimuli throughout the developmental process and that repeated exposure to stroking stimuli for more than 4 weeks induced positive emotion in rats in response to stroking stimuli. Consistent with the results of the present study showing that repetitive stroking stimuli by a single experimenter induced 50-kHz calls, tactile stimuli by a familiar experimenter, but not those by an unfamiliar experimenter, have been shown to induce 50-kHz vocalization^8,43,44^.

In order to examine the specificity of effects of stroking stimuli, ultrasonic vocalizations during basal, holding, and stroking conditions were recorded. Rats emitted a larger number of 50-kHz calls during stroking than during the basal condition, particularly in the S3–10 group. Stroking stimuli at a speed of 1–10 cm/s have been shown to activate unmyelinated low-threshold mechano-sensitive C-tactile afferents to induce a pleasant sensation^45,46^. These findings suggest that after receiving repeated stroking stimuli, tactile sensation of stroking stimuli induced positive emotion in rats. In addition, the number of FM and non-FM calls during stroking was increased in the S3–6, S7–10, and S3–10 groups. The number of FM 50-kHz calls has been shown to be increased in socially positive emotional states^24^. Among the FM 50-kHz calls, the numbers of complex, trill, and inverted U calls increased during stroking in the present study. Trill calls have been shown to be increased after tickling stimuli, which are considered to be strong reward stimuli for rats^47^. On the other hand, in the present study, the most frequent subtype of syllables emitted was the complex type. The function of the complex type of vocalization remains to be clarified. Further research is needed to clarify the specific role of each syllable in rats.

In the present study, animals that received repeated stroking stimuli during the adolescent period showed preference toward the experimenter’s hand and showed following behavior towards the experimenter’s hand. In addition, in the S3–6, S7–10 and S3–10 groups, locomotor activity was increased in the box conditioned with stroking stimuli, though the times spent staying in the box did not significantly change. Consistent with these findings, locomotor activity has been shown to be increased in a place conditioned with amphetamine in an amphetamine-induced conditioned place preference test^48,49^. All of these findings suggest that stroking stimuli have reward values in rats that have received repeated stroking stimuli during the adolescent period. Interestingly, the numbers of 50-kHz calls during the stroking condition were positively correlated with the following time in the following behavior test and the preference ratio in the CPP test (ratio of time spent in the I.N.P. box conditioned with stroking stimuli to time spent in the box before conditioning) (Supplementary Table 2). The findings indicated that rats that showed a larger number of 50-kHz calls in response to stroking showed more affiliative behaviors toward the experimenter.

During the development of domestication processes of wild animals, affiliative behaviors toward humans have often been shown to be associated with reduced levels of anxiety. For example, during selection processes of tame rats from wild-caught gray rats, rats showing no aggressive behavior toward humans have been selected. These rats showed a low level of anxiety-related behavior in an open field test and a startle-response test^50,51^, suggesting that affiliative responses toward humans are correlated with lower stress responses. In the present study, neither the time spent in the center area nor the immobile time in an open field test were significantly different among N3–10 control and stroking groups. Locomotor activity in the stroking groups was also not significantly different compared with that in the non-stroking N3–10 control group, although that in the S3–10 group was significantly higher than that in the S3–6 or S7–10 group. These findings suggest that stroking stimuli in the developmental period induced affiliative behavior towards humans but did not significantly change anxiety-related behavior. On the other hand, there was a positive correlation between the number of 50-kHz calls during stroking and center time in an open field test and there was a negative correlation between the number of 50-kHz calls and immobile time, being consistent with the possible negative relationship between affiliative behavior and anxiety-related behavior. Further studies including investigation of other anxiety-related indexes need to be performed to clarify the relationship between affiliative behavior and anxiety-related behavior.

Gentle tactile stimuli have been shown to increase positive emotional behaviors^36,52,53^ and to facilitate affiliative behavior^28^. For example, early tactile stimuli such as licking/grooming behavior toward their pups have been shown in rodents to modulate social behaviors of the pups in adulthood^54^. On the other hand, tactile stimuli have been shown to activate oxytocin systems. Stroking stimuli increased urinary oxytocin concentration in dogs^37^. Our study revealed that hypothalamic oxytocin neurons, especially those located in the caudal PVN, were activated by stroking stimuli after repeated stroking experiences, being consistent with the results of our previous study^29^. Oxytocin plays an important role in the formation of an affiliative relationship^28,55–57^. Oxytocin neurons in the PVN have been shown to project to the ventral tegmental areas, periaqueductal gray, nucleus tractus solitarius, and spinal cord^58–60^. These brain areas are associated with processing for reward value^61^. From these findings, it is tempting to speculate that stroking stimuli during the post-weaning period facilitate the formation of an affiliative relationship and that once an affiliative relationship starts to form, stroking stimuli strengthen the affiliative relationship via activation of oxytocin neurons in the caudal PVN.

The adolescent period of rats has been divided into early adolescent period (a peripubertal phase of sexual maturation, postnatal 3–5 weeks in rats), mid-adolescent period (postnatal 5–7 weeks) and late adolescent period (postnatal 7–9 weeks)^62–64^. Plasma testosterone levels, which affect social behavior, start to increase gradually in the mid-adolescent period and steeply in the late adolescent period. In this study, the effects of stroking stimuli during the first half (3–6 weeks of age), second half (7–10 weeks of age) and whole period (3–10 weeks of age) of adolescence were examined to identify the critical period for the induction of an affiliative response toward humans. Social experiences during the early adolescent period are thought to be critical for social and cognitive development^64^. Dogs have a critical period for socialization to humans. Experiences during the socialization period of dogs have been shown to greatly influence fearfulness and aggressiveness to humans^65–67^. Early training of puppy dogs and early handling of lambs have been reported to induce positive reactions toward humans^68,69^. In rats, social isolation in developmental periods induces impairments in various social behaviors^70^. From these reports, we hypothecated that there is a critical period for induction of affiliative responses toward humans. The S3–6, S7–10, and S3–10 groups spent a longer time in the experimenter’s hand area than in the bottle area, showed following behavior toward the experimenter’s hand, emitted 50-kHz calls more frequently during stroking stimuli than in the basal condition, and exhibited higher locomotor activity in the box previously conditioned with stroking stimuli. These findings suggest that rats that received post-weaning stroking stimuli showed a tendency for positive reactions toward stroking stimuli or the experimenter. However, there were significantly higher preference scores in the social preference test in the S3–6 and S3–10 groups than in the N3–10 control group, and the preference ratio of time in the CPP test was significantly higher in the S3–6 group than in the N3–10 group. The numbers of 50-kHz USVs during the following test in the S3–6 and S3–10 groups were significantly larger than the number in the N3–10 control group. These findings are consistent with the view that the first post-weaning 4-week period is important for induction of affiliative responses toward humans, although boundaries of the critical period are not clear.

In summary, our results indicate that post-weaning stroking procedures facilitate affiliative responses in rats possibly via activation of oxytocin neurons in the caudal PVN. Our results provide an important animal model for elucidation of affiliative relationships between humans and other animals, which should contribute to the enhancement of health of both humans and companion animals.

## Supplementary information

Supplementary figures, legends and tables.

## Data Availability

All data analyzed during this study are included in this published article and its Supplementary Information file. The detailed data sets are available on reasonable request.
