# Supplementary material for: Gentle stroking stimuli induce affiliative responsiveness to humans in male rats
Source: Sci Rep. 2020 Jun 4;10:9135. doi: 10.1038/s41598-020-66078-7 (PMC7272613; doi:10.1038/s41598-020-66078-7)
Supplement: Supplementary file 1 — Supplementary figures, legends and tables. [file 41598_2020_66078_MOESM1_ESM.docx]

**Supplementary information**

**Gentle stroking stimuli induce affiliative responsiveness to humans in male rats.**

Shota Okabe, Yuki Takayanagi, Masahide Yoshida, Tatsushi Onaka^*^,

Supplementary Fig. 1

Supplementary Fig. 2

Supplementary Table 1

Supplementary Table 2

**Supplementary Fig. 1**

Numbers of vocalizations of each syllable subtype in the N3-10 (A), S3-6 (B), S7-10 (C), and S3-10 (D) groups. Statistical *P* values calculated from comparisons among intra-syllable subtypes are shown in the figure. Statistical results of comparisons among intra-syllable subtypes and inter-syllabble subtypes are as follows. Repeated measures two-way ANOVA followed by Holm’s test was performed.

In the N3-10 group, repeated measures two-way ANOVA showed a significant effect of syllable subtype and a significant effect of condition but no significant interaction. Post-hoc analysis showed that the number of calls in the stroking condition was significantly larger than that in the basal or holding condition. However, there was no significant difference in the number of vocalizations among syllable subtypes.

In the S3-6 group, ANOVA showed significant effects of syllable subtype and condition and a significant interaction. In the basal condition, the number of flat call was significantly larger than the numbers of complex (*P* = 0.0063), upward (*P* = 0.0063), split (P = 0.0063), step down (*P* = 0.0063), multiple step (*P* = 0.0063), trill (*P* = 0.0063), inverted U (*P* = 0.0063), composite (*P* = 0.0063), and downward calls (*P* = 0.0079, post-hoc Holm’s test). In the stroking condition, the number of short calls was significantly larger than the numbers of split (*P* = 0.0322), composite (*P* = 0.0361), and downward calls (*P* = 0.0479, post-hoc Holm’s test). The number of step down calls in the stroking condition was significantly larger than that in the basal or holding condition (stroking versus basal condition, *P* = 0.019; stroking versus holding condition, *P* = 0.019, post-hoc Holm’s test).

In the S7-10 group, ANOVA showed significant effects of syllable subtype and condition and a significant interaction. In the basal condition, the number of flat calls was significantly larger than the numbers of complex (*P* = 0.0063), upward (*P* = 0.0063), downward (*P* = 0.0063), split (*P* = 0.0063), step up (*P* = 0.0063), step down (*P* = 0.0063), multiple step (*P* = 0.0063), trill (*P* = 0.0063), inverted U (*P* = 0.0063), and composite calls (*P* = 0.0063, post-hoc Holm’s test). In the stroking condition, the number of short calls was significantly larger than the numbers of split (*P* < 0.0001), composite (*P* < 0.0001), downward (*P* < 0.0001), step down (*P* < 0.0001), inverted U (*P* < 0.0001), upward (*P* < 0.0001), multiple step (*P* < 0.0001), step up (*P* = 0.0001), trill (*P* = 0.0013), and flat calls (*P* = 0.0226, post-hoc Holm’s test). In addition, in the stroking condition, the number of complex calls was significantly larger than the numbers of split (*P* = 0.0240) and composite calls (*P* = 0.0297, post-hoc Holm’s test). The numbers of complex (*P* = 0.014), multiple step (*P* = 0.0352), trill (*P* = 0.0411), inverted U (*P* = 0.0172), and short calls (*P* = 0.0199, post-hoc Holm’s test) in the stroking condition were significantly larger than the numbers of corresponding calls in the basal condition.

In the S3-10 group, ANOVA showed significant effects of syllable subtype and condition and a significant interaction. In the basal condition, the number of flat calls was significantly larger than the numbers of complex (*P* < 0.0001), upward (*P* < 0.0001), split (*P* < 0.0001), step up (*P* < 0.0001), multiple step (*P* < 0.0001), trill (*P* < 0.0001), downward (*P* < 0.0001), inverted U (*P* < 0.0001), composite (*P* < 0.0001), and step down calls (*P* = 0.0025, post-hoc Holm’s test). In the holding condition, the numbers of flat and short calls were significantly larger than the numbers of split, step down, composite, downward, multiple step, inverted U, upward, trill, step up, and complex calls (flat or short versus split, step down or composite, *P* < 0.0001; flat versus downward or multiple step, *P* < 0.0001; short versus downward or multiple step, *P* = 0.0001; flat or short versus inverted U or upward, *P* = 0.0001; flat versus trill, *P* = 0.0001; short versus trill, *P* = 0.0002; flat versus step up, *P* = 0.0031; short versus step up, *P* = 0.0038; flat versus complex, *P* = 0.0076; short versus complex, *P* = 0.0094, post-hoc Holm’s test). In the stroking condition, the number of short calls was significantly larger than the numbers of split (*P* < 0.0001), composite (*P* < 0.0001), downward (*P* < 0.0001), multiple step (*P* < 0.0001), step down (*P* < 0.0001), inverted U (*P* < 0.0001), upward (*P* < 0.0001), step up (*P* = 0.0002), trill (*P* = 0.0011), and flat calls (*P* = 0.0121, post-hoc Holm’s test). The number of complex calls was significantly larger than the numbers of split (*P* = 0.0027), composite (*P* = 0.0032), downward (*P* = 0.0096), multiple step (*P* = 0.0113), step down (*P* = 0.0130), inverted U (*P* = 0.0130), and upward calls (*P* = 0.0253). The numbers of complex (*P* = 0.0078), step up (*P* = 0.013), trill (*P* = 0.0243), inverted U (*P* = 0.0134), flat (*P* = 0.0365), and short calls (*P* = 0.0107, post-hoc Holm’s test) in the stroking condition were significantly larger than the numbers of corresponding calls in the basal condition. The numbers of complex (*P* = 0.0122), step up (*P* = 0.0492), trill (*P* = 0.0351), inverted U (*P* = 0.0309), and short calls (*P* = 0.0136) in the stroking condition were significantly larger than those in the holding condition. Finally, the numbers of complex (*P* = 0.0122), step up (*P* = 0.0019), and short calls (*P* = 0.0107, post-hoc Holm’s test) in the holding condition were significantly larger than those in the basal condition. †, *P* < 0.01; *, *P* < 0.05, post-hoc Holm’s test. Error bars denote standard error of the mean.

**Supplementary Fig. 2**

Numbers of oxytocin-immunoreactive (-ir) neurons in the caudal PVN (A), rostral PVN (C), BNST (E), and SON (G). Numbers of c-Fos-immunoreactive (-ir) neurons in non-oxytocin neurons in the caudal PVN (B), rostral PVN (D), BNST (F), and SON (H). The numbers of oxytocin-ir cells in the caudal PVN, rostral PVN, BNST, and SON were not significantly different among the four groups. The numbers of non-oxytocin-ir neurons expressing immunoreactivity of c-Fos protein were significantly increased after stroking stimuli in the caudal PVN, rostral PVN, and BNST but not in the SON. †, *P* < 0.01. Error bars denote standard error of the mean.

|  | **Oxytocin-ir neurons expressing c-Fos protein (%) in caudal PVN** | |
| --- | --- | --- |
| **Behaviors** | **r** | **P value** |
| Moving distance in the open field test | 0.0344 | 0.8791 |
| Total center time in the open field test | 0.1825 | 0.4163 |
| Immobile time in the open field test | 0.0023 | 0.9920 |
| Preference score in the social preference test | 0.3202 | 0.1463 |
| Following time in the following behavior test | 0.2355 | 0.2915 |
| Number of 50-kHz calls during the following behavior test | 0.0721 | 0.7500 |
| Number of 50-kHz calls during the stroking condition | 0.4762 | 0.0251 * |
| Preference ratio in the CPP  (Time spent staying in the I.N.P. box during the post-conditioning/ Time spent staying in the I.N.P. box during the pre-conditioning) | 0.2174 | 0.3311 |
| Preference ratio in the CPP  (Locomotion distance in the I.N.P. box during the post-conditioning/ Locomotion distance in the I.N.P. box during the pre-conditioning) | 0.4986 | 0.0182 * |
| Preference ratio in the CPP  (Locomotion distance in the I.P. box during the post-conditioning/ Locomotion distance in the I.P. box during the pre-conditioning) | 0.2818 | 0.2040 |

**Supplementary Table 1**

Spearman’s correlations between percentages of oxytocin-ir neurons expressing c-Fos protein in the caudal PVN and each behavioral parameter. Data for rats in all experimental groups (N3-10, S3-6, S7-10, and S3-10) that were assigned to the stroking condition before perfusion were used for statistical analysis (n = 22). *, *P* < 0.05.

|  | **Number of 50-kHz calls during the stroking condition** | |
| --- | --- | --- |
| **Behaviors** | **r** | **P value** |
| Moving distance in the open field test | 0.4154 | 0.0050 † |
| Total center time in the open field test | 0.4512 | 0.0021 † |
| Immobile time in the open field test | -0.4655 | 0.0015 † |
| Preference score in the social preference test | 0.1024 | 0.5083 |
| Following time in the following behavior test | 0.4577 | 0.0018 † |
| Number of 50-kHz calls during the following behavior test | 0.5620 | < 0.0001 † |
| Preference ratio in the CPP  (Time spent staying in the I.N.P. box during the post-conditioning/ Time spent staying in the I.N.P. box during the pre-conditioning) | 0.4938 | 0.0007 † |
| Preference ratio in the CPP  (Locomotion distance in the I.N.P. box during the post-conditioning/ Locomotion distance in the I.N.P. box during the pre-conditioning) | 0.1742 | 0.2582 |
| Preference ratio in the CPP  (Locomotion distance in the I.P. box during the post-conditioning/ Locomotion distance in the I.P. box during the pre-conditioning) | -0.2394 | 0.1176 |

**Supplementary Table 2**

Spearman’s correlations between the number of 50-kHz calls during the stroking condition (data in Fig. 4A) and behavioral parameters in each behavioral test. Data for all animals (N3-10, S3-6, S7-10, and S3-10 groups) were used for statistical analysis (n = 44). †, *P* < 0.01.
